# Comparing the Effectiveness of a Virtual Toxicology Escape Room at Two Emergency Medicine Residencies

**DOI:** 10.7759/cureus.11262

**Published:** 2020-10-30

**Authors:** Alexis L Cates, James Krueger, Serge-Emile Simpson, Megan Stobart-Gallagher

**Affiliations:** 1 Emergency Medicine/Medical Toxicology, Einstein Medical Center Philadelphia, Philadelphia, USA; 2 Emergency Medicine, Thomas Jefferson University Hospital, Philadelphia, USA

**Keywords:** toxicology, medical education, virtual academics, emergency medicine

## Abstract

Introduction

Physical distancing guidelines during the coronavirus disease 2019 (COVID-19) pandemic forced medical residency programs to move a large portion of required didactics to virtual settings. Toxicology, a core component of emergency medicine (EM) education, was forced to adapt to similar constraints. An in-person escape room style puzzle was modified to a virtual format for educational purposes, and shared with and evaluated by two different residency programs.

Materials and methods

A virtual escape room, “Escape the Toxin: Online!” was created to test knowledge of toxicologic ingestion and antidote utilizing Google Forms and delivered using Zoom teleconference software to two EM residency programs in the Philadelphia region. After small groups completed the gamified activity, their scores were calculated and they completed an anonymous evaluation.

Results

Residents at the program where a Medical Toxicology fellowship is located found the virtual escape room to be more effective and enjoyable compared to the second program. Despite some differences in perceived effectiveness, the majority of participants were able to correctly solve the puzzle and get to the antidote.

Conclusion

The majority of learners who participated from both residencies agreed that they would recommend this virtual program to other EM residents.

## Introduction

Toxicology is a core component of emergency medicine (EM) resident education. In an effort to promote engagement with residents, the Division of Medical Toxicology at Albert Einstein Healthcare Network in Philadelphia, PA had previously created an immersive escape room experience titled “Escape the Toxin.” This team-based puzzle involved a geographical maze within departmental office space to find an antidote for a given toxidrome. As educators, we sought to share and replicate it within other regional residency programs. However, in an unprecedented era of physical distancing due to the coronavirus disease 2019 (COVID-19) pandemic, many residency programs found themselves planning for virtual educational content to replace in-person didactics. Adaptations to this new style of learning included live virtual conferences and lectures, in addition to increased asynchronous online learning [1-3]. Therefore, we had to find yet a new way to provide engaging didactic content to our learners in toxicology.

The original “Escape the Toxin” innovation was created as a result of gamification becoming increasingly popular in medical education. Gamification in this sense is the incorporation of game elements into didactics to increase engagement among participants. Gamification has shown effectiveness in improving knowledge, skills, and satisfaction compared to traditional lectures [4-6]. Learner satisfaction with gamification also seems to be relatively high. In a multi-national systematic review, gamification for health professions was, in many cases, found to have relatively high satisfaction ratings when compared to the controls of traditional learning or other digital education modalities [4]. The systematic review identified that much of the findings were of low quality due to inconsistency across the types of learning modalities compared. Further targeted research is necessary to identify that gamification allows educational goals to be reached as good or better than other modalities of learning. However, this denoted a promising finding: experiential learning through gamification as a virtual or digital modality is likely just as effective as traditional learning. Part of our inspiration to develop "Escape the Toxin" was drawn from "The Toxiscape Hunt," a didactic activity published by physician educators at the University of California at Irvine in the Department of Emergency Medicine [7]. Though we had a case-based approach with a single toxidrome, their puzzles also sought to bring a team of medical students and residents together to learn multiple toxicological concepts and associated patient care. 

In an effort to continue resident education throughout the COVID-19 pandemic and add to the growing online medical education movement, we utilized Google Forms to develop a virtual version, “Escape the Toxin: Online!” Though the virtual version did not include physical locations or cryptex puzzle lockboxes containing a clue, it retained the same underlying theme: determine the antidote to the toxidrome presented. After an initial description of the patient, signs, symptoms, and vital signs, learners were then given multiple intermingled toxicology challenges. The ultimate goal of the activity was to pick the correct mechanism of action for the antidote to “escape the toxin.”

## Materials and methods

The challenge was incorporated into weekly didactics at two regional EM residency programs that had successfully migrated onto a digital platform. The first program used the virtual escape room as either a live online activity during didactics or as an optional asynchronous activity, while the second program provided it as part of the required weekly didactics. During both sessions, the Zoom teleconferencing platform was utilized with breakout rooms. These rooms allowed for the task to be completed in small groups, providing an opportunity for a team-based approach to solve the challenges mimicking a live escape room.

**Figure 1:**
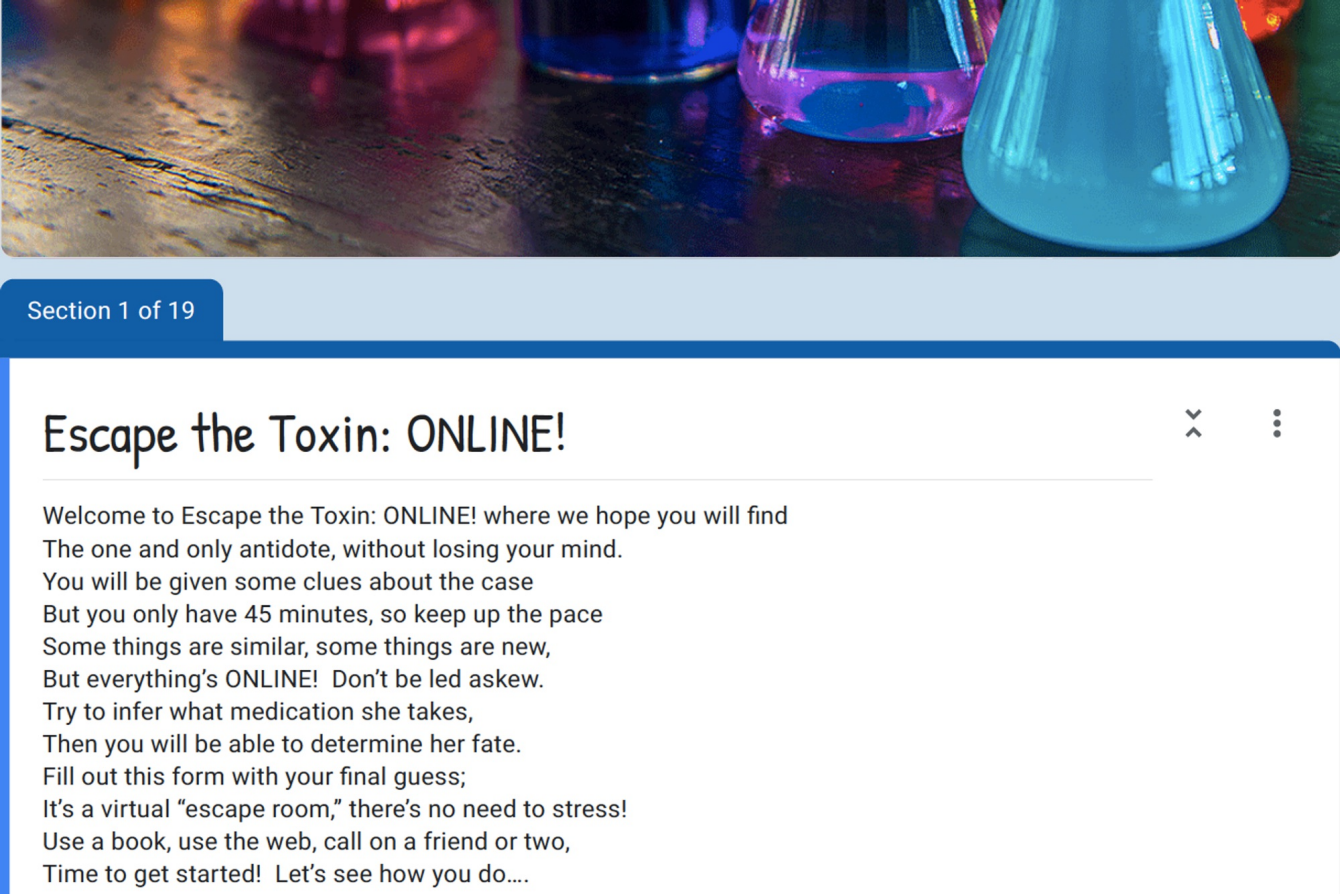
Introduction to Escape the Toxin: Online! Image created by authors.

For each iteration, the entire group met in the main room before being divided into virtual breakout rooms of no more than six or seven participants. They received a brief introduction and the link to the Google Form containing the virtual escape room (Figure 1). With the first program, the groups were assigned randomly by the Zoom software. At the second program, the groups were assigned manually by a faculty facilitator to aid in keeping track of teams in preparation for potential technological glitches. Participants had a maximum of 45 minutes in which to complete the challenge.

**Figure 2:**
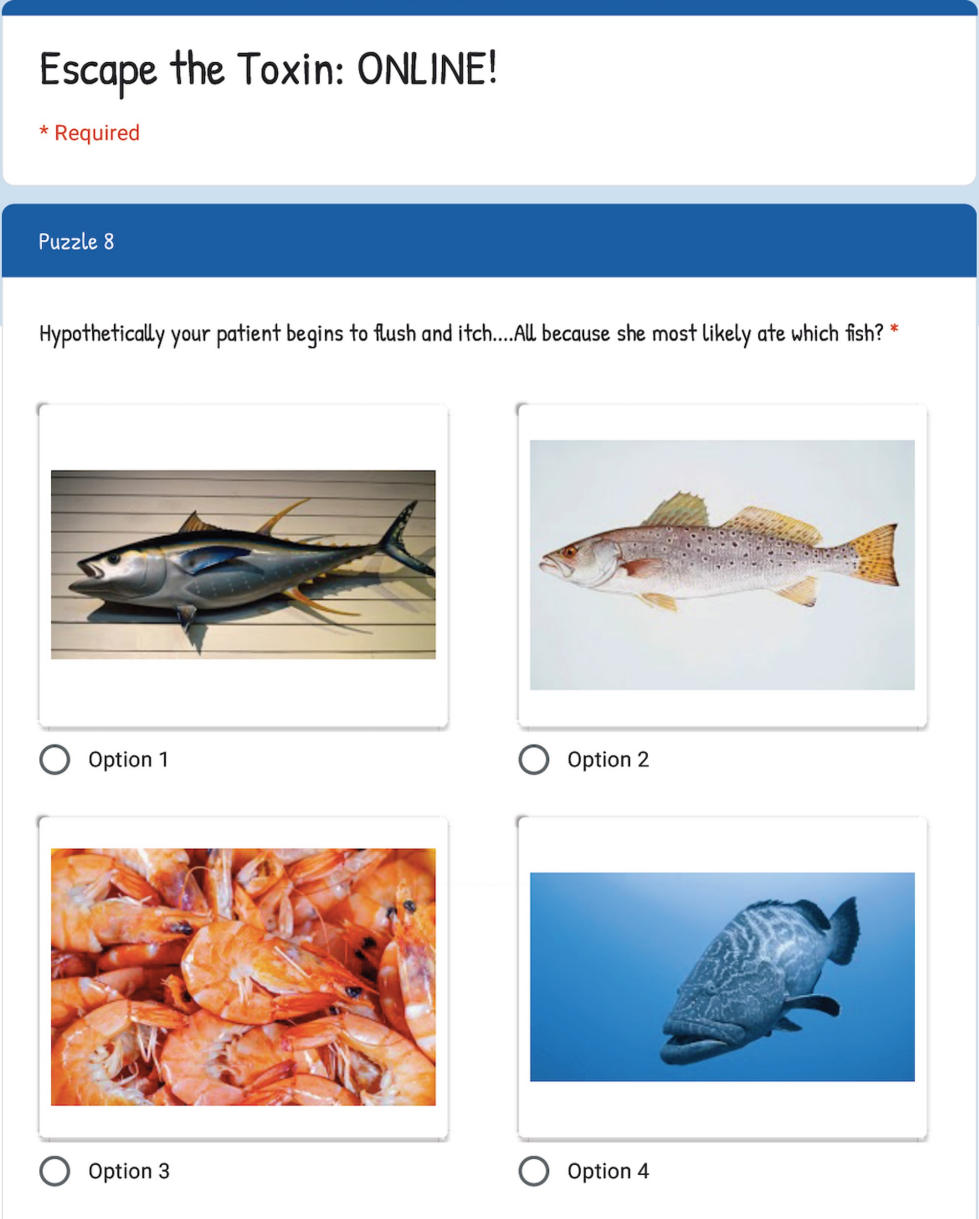
Example of puzzle during Escape the Toxin: Online! Image created by authors.

To move forward through the Google Form, participants were required to answer all of the questions. In order to preserve flow, the answers did not have to be correct to move to the next puzzle, and participants were not notified in real time if their answer was incorrect. However, in order to accomplish the ultimate goal of picking the correct antidote (located in the final puzzle), the participant needed to have identified the toxidrome described in the initial description of the case. The remainder of the questions and puzzles all had rhyming stems to apply gamification mechanics to mimic an in-person escape room experience. A variety of question types were utilized, including anagrams, fill-in-the-blank, multiple-choice or short answer (Figure 2). The puzzles were developed to be challenging and thought-provoking, especially since rhyming mechanics were used. Much like an in-person escape room, brainstorming, using available resources, thinking out loud and teamwork were required to decipher the intent of the rhyme. However, the puzzles always referenced the toxidrome and antidote pair, including signs, symptoms, diagnostic findings, alternative therapies, and additional treatments by the antidote. The final question related back to the mechanism of action of the proposed antidote that matched with the original poisoning. 

At the end of the activity, a short three-question survey was embedded to obtain feedback about the learner’s attitude toward this modality for presenting toxicology content, the appropriateness of the time allotted, and whether the participant would recommend the activity to other EM residents. Responses to these Likert-scale questions were required to complete the game. After the live session, participants were invited to debrief about the puzzles and the case itself. The responses to the Google Form were reviewed. The top learners were identified, and considered to have “escaped the toxin” or “passed the case” if they met the following criteria: selected the correct mechanism of action of the antidote, had the most correct answers from the puzzles, and completed the activity within the allotted 45 minutes.

## Results

“Escape the Toxin: Online!” is an innovative virtual activity that was successfully integrated into individual didactic sessions by two regional EM residency programs. With the gamification feel, it brought EM residents together in a fun, relaxed environment to use each other and their research skills to learn about a toxidrome and the associated antidote that they may encounter over their careers. 

Across the two iterations, 46 residents participated. The first program had 17 participants from a postgraduate year 1-4 (PGY1-4) residency with a Medical Toxicology fellowship at their institution. The second program had 29 participants from a PGY1-3 residency without an associated fellowship. Overall, 54% (25 out of 46) of the resident participants felt that this activity was helpful or very helpful towards learning toxicology. The participants at both programs all completed the activity in the time allotted. At the first program, 88% (15 out of 17) of participants completed the task by identifying the correct mechanism of action of the antidote and 95% (28 out of 29) of participants were correct at the second program.

Data from the two programs was not only collated for total effectiveness but also compared, as shown in Table 1. At the first program, 94% (16 out of 17) of participants found the activity helpful or very helpful as a teaching tool for the study of toxicologic concepts with only 31% (9 out of 29) of participants feeling the same at the second program. Approximately 7% (two out of 29) of participants at the second program did not find this activity to be helpful and two did not answer the question but rather addressed their concerns with the activity via free text. One comment stated they wished they “had teammates,” but it was not immediately clear if this person was participating away from the original teleconference, as a faculty facilitator had manually divided participants into groups. A second comment highlighted the participant’s confusion of “what the question being asked was.” 

**Table 1:** Comparison of Individual Residency Programs Performance and Evaluations

	First iteration N=17 (with toxicology fellowship)	Second iteration N=29 (without toxicology fellowship)
% respondents who felt there was adequate time for activity	65%	72%
% respondents who felt there was too much time for activity	29%	10%
% respondents who felt activity was helpful or very helpful	94%	31%
% respondents who would recommend activity to other EM residents	100%	41%
% respondents who completed activity in time allotted	100%	100%
% respondents who chose correct mechanism of action of antidote	88%	95%

Overall, 58% of the total participants agreed or strongly agreed that they would recommend this activity to other EM residents as a way to learn toxicologic concepts. When comparing the two programs, the first iteration found 100% of participants to be in agreement, but only 41% (12 out of 29) at the second program. Seventeen percent (five out of 29) of participants felt disagreement or strong disagreement that they would recommend this activity to other colleagues. 

## Discussion

Interestingly, the majority of the residents who felt this activity was helpful came from the residency program with an associated Medical Toxicology fellowship. The duties of a medical toxicology fellow and faculty usually extend from bedside clinical consultations to a wide variety of lectures and on-the-job teaching [8]. This regular interaction in a variety of learning environments, including a previous in-person escape room activity, likely contributed to knowledge and comfort of handling a poisoned patient's case. 

The program without a Medical Toxicology fellowship had been previously exposed to gamification in their weekly didactics, but may have been fatigued of this concept. This could have contributed to less satisfaction with the activity at the time it was delivered. Adequate spacing of these innovative didactic sessions may improve engagement. The participants may not have known what to expect from this particular type of gamification. One of the free text comments from a participant at the second program alluded to the questions being somewhat vague. In an in-person escape room style puzzle, many of the clues are intentionally vague to lead the team to the correct answer with real-time brainstorming and interaction with teammates. After analyzing these results, we recommend an expanded explanation of the activity for programs that have not experienced an in-person escape room style puzzle for future activities.

The presence of a Medical Toxicology fellowship is also a huge benefit for continuity and sustainability of the virtual program. The creation of this virtual learning modality may be more time-consuming and require more knowledge and effort than traditional learning methods [9]. It would likely not be sustainable where there are no Medical Toxicology fellows or faculty to devote the time and resources to the development and implementation of this time of the project.

Though there is a question as to the success of resident engagement among the two iterations, 88% of the first group and 95% of the second group ultimately answered the final question correctly. This indicated an overall understanding of the presented toxidrome, the diagnosis in the case and found the antidote in question. It also likely indicates decent engagement in the activity.

For EM programs that have not participated in a similar activity or an in-person escape room, a thorough explanation of the concept is warranted to improve resident participation and experience. Lastly, an anonymous evaluation following a formal debriefing may be more reflective of participant engagement and thoughts on the success of the activity, and should be considered in future projects.

## Conclusions

Virtual medical education has been widely utilized due to physical distancing requirements during the COVID-19 pandemic and is likely to continue beyond this public health emergency. Constraints of time and availability of educators make virtual learning an appealing option to many EM programs. EM programs should familiarize their residents with different types of virtual learning, including gamification concepts such as “Escape the Toxin: Online!” Despite some differences in perceived effectiveness, the majority of participants in "Escape the Toxin: Online!" were able to correctly solve the puzzle and choose the correct antidote. Further study is needed to identify why an EM program with a Medical Toxicology fellowship may find increased satisfaction, though a plausible explanation is resident comfort with Medical Toxicology fellows and faculty. However, adequate explanation of the virtual escape room concept prior to implementation in a program’s curriculum may improve engagement, satisfaction, and ultimately, retention of information.
